# Host–parasite interactions of rodent hosts and ectoparasite communities from different habitats in Germany

**DOI:** 10.1186/s13071-021-04615-7

**Published:** 2021-02-17

**Authors:** Anna Obiegala, Leonie Arnold, Martin Pfeffer, Matthias Kiefer, Daniel Kiefer, Carola Sauter-Louis, Cornelia Silaghi

**Affiliations:** 1 Institute of Animal Hygiene and Veterinary Public Health, University of Leipzig, Leipzig, Germany; 2 Comparative Tropical Medicine and Parasitology, Ludwig-Maximilians-Universität München, Munich, Germany; 3 Institute of Epidemiology, Friedrich-Loeffler-Institut, Federal Research Institute for Animal Health, Greifswald, Riems Germany; 4 Bavarian State Collection of Zoology, Munich, Germany; 5 Institute of Infectology, Friedrich-Loeffler-Institut, Federal Research Institute for Animal Health, Greifswald, Riems Germany

**Keywords:** Host–parasite interaction, Ectoparasites, Ticks, Rodents, Fleas, Mites, *Myodes glareolus*, *Apodemus* spp., *Ixodes ricinus*

## Abstract

**Background:**

Small mammals are important maintenance hosts of ectoparasites as well as reservoir hosts for many arthropod-borne pathogens. In Germany, only a few studies have investigated ectoparasite communities on small mammals in their natural habitats. The aim of this study was to assess the species diversity and parameters influencing the mean intensity and prevalence of macroscopically visible ectoparasites, such as fleas, predatory mites and ticks.

**Methods:**

A total of 779 small mammals and 3383 ticks were available from earlier investigations for the data analysis of the current study from three differently structured study sites. In addition, fleas and predatory mites were collected from the captured rodents and taxonomically identified. Regression analyses were conducted on the group (ticks/mites/fleas) and species levels using hurdle models for the abundance of ectoparasite groups and a negative binomial model for the abundance of species.

**Results:**

Nearly 90% of the small mammals analyzed were infested with ectoparasites, with an average of 7.3 specimens per host. Hosts were infested with up to six species of ectoparasites simultaneously. In total, 12 flea, 11 mite and three tick species were detected. Ticks were more prevalent than fleas or mites, with > 80% of the hosts in urban and forest areas hosting ticks and around 60% of hosts presenting fleas, and only 20–40% of hosts presenting mites. Polyparasitism had a statistically significant influence on the prevalence of the investigated tick, mite and flea species, with odds ratios of > 1.0. Trapping location, season and host characteristics had significant influences on some—but not all—of the investigated species.

**Conclusions:**

The diversity of flea species was unexpectedly high and higher than that reported in comparable studies, which can be explained by the differently structured habitats and regions examined in this study. Polyparasitism was a key influencing factor and had a positive effect on the prevalence and/or abundance of the predominant tick, flea and mite species occurring on small mammals. Season, trapping location, host species and sex of the host species also had an influence on the prevalence and mean intensity of certain, but not all, ectoparasite species.
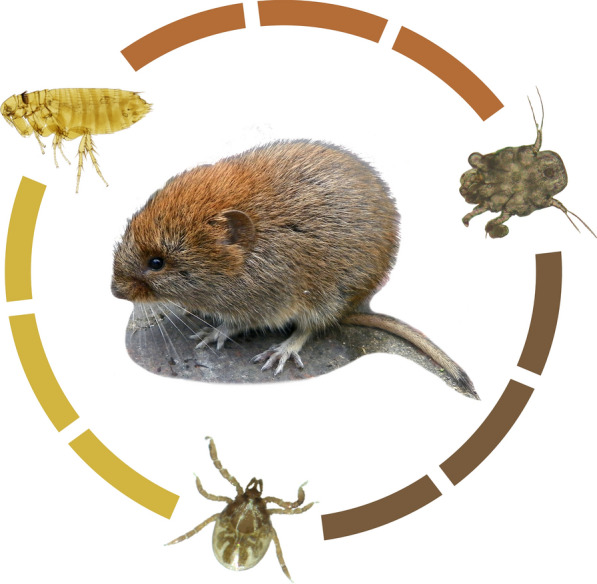

## Background

Small mammals are important reservoir hosts for the maintenance of developmental and adult stages of ectoparasites. Additionally, small mammals serve as reservoirs for many different arthropod-borne pathogens and may thus play an important role in the maintenance and distribution of these pathogens [1]. In central Europe, the most common small mammal species found in the wild in woodlands are *Myodes glareolus* (bank vole) and *Apodemus flavicollis* (yellow-necked mouse) [2]. A previous study on ectoparasites occurring on small mammals in Germany reported 63 different ectoparasite species on six common small mammal species, with an average ectoparasite intensity of 16 specimens per small mammal [3]. Thus, the distribution and density of small mammal populations may have a direct impact on ectoparasite populations and further on the spread of arthropod-borne pathogens. In central Europe, *M. glareolus* and *A. flavicollis* are reservoirs for many viral, bacterial and parasitic arthropod-borne agents, such as tick-borne encephalitis virus (TBEV), *Borrelia burgdorferi* (*s.s.*) and *Babesia microti* [4–6]. A strong correlation between *Dermacentor reticulatus* ticks and *M. glareolus*, in comparison to that between *D. reticulatus* and *A. flavicollis*, has been reported [7]. *Dermacentor reticulatus* is suggested to be the main vector for *Rickettsia raoultii* [8], and a sevenfold higher prevalence for *R. raoultii* was observed in *M. glareolus* infested with *D. reticulatus *than in *M. glareolus* not infested with *D. reticulatus* [7]. Another recent study showed the occurrence of *Rickettsia* spp. in 11 different ectoparasite species, such as fleas, ticks and predatory mites [9]. The composition, density and abundance of ectoparasite communities may influence the prevalence rates of arthropod-borne pathogens in small mammal hosts, as suggested by the wide ranges in prevalence rates of several arthropod-borne pathogens that have been reported in different European countries [10, 11]. Although *A. flavicollis* and *M. glareolus* mainly inhabit woodlands, they can also be found in proximity to human settlements, such as in gardens, basements and parks [12]. Nevertheless, to date, small mammals play a subordinate role in public health surveillance programs in Germany. In the past decade, only a few studies examing the relationship between small mammals and the abundance and variety of ectoparasites, such as ticks, fleas and predatory mite communities, have been carried out in Europe [3, 9, 13, 14]. Less recent studies are also scarce [15, 16]. To our knowldege, only one study to date has investigated the variety of ectoparasite communities on small mammals in Germany [3].

 Therefore, the aims of this study were: (i) to determine the species diversity of macroscopically visible ectoparasites, such as fleas, predatory mites and ticks, on various small mammal hosts from differently structured sites in Germany; (ii) to investigate the effect of host species, trapping location and season on the infestation of small mammals with ectoparasites; and (iii) to assess the factors influencing the abundance of the hard tick species *Ixodes ricinus*, the mite species *Laelaps agilis* and the flea species *Megabothris turbidus*,* Ctenophthalmus agyrtes*, *C. congener congener,* and *C. bisoctodentatus* on mice and voles.

## Methods

### Small mammal and arthropod collections

A total of 779 small mammals and 3383 ticks, all available from previous investigations (Additional file 1: Tables S1–S4) [7, 17, 18], were included in the data analysis of the present study. Four animals, mentioned in the category “others”, with undetermined species or species other than mice or voles, were not included in the present analysis. Small mammal trapping took place at three differently structured locations. Two of those sites were located in southern Germany: the first was a small urban park in the city of Regensburg (49°00′55.72″N, 12°05′08.89″E) (previously called R1), and the second was a sylvatic large forest in Bavaria (48°06′36.42″N, 10°34′33.40″E) (previously called T). The third site (previously called S) was a renatured recreational area near Leipzig, Saxony that was subdivided into three parts (51°15′32.2″N, 12°21′02.5″E, 51°17′01.3″N, 12°21′00.6″E and 51°26′97.2″N, 12°32′25.6″E, respectively) and highly frequented by visitors. Small mammals were collected with Sherman© live animal traps (H.B. Sherman Traps, Inc., Tallahassee, FL, USA) at all three study sites (official permits: AZ 36.11-36.45.12/4/12-001; 55.1-8646.4-140; 55.1-8646-2/30). Traps were baited with apple slices and placed for two consecutive nights per month at each site and checked daily twice. Collected rodents were anaesthetized with CO_2_ and euthanized by cervical dislocation. Detailed descriptions on the study sites and trapping procedures are provided in earlier publications [7, 17, 18]. Small mammal species were identified using taxonomic keys [19] as well as by using conventional PCR targeting the *cytochrome b* gene, yielding an amplicon of 354 bp for 15 randomly selected wood mice, 14 bank voles, 23 yellow-necked mice and all shrews, common voles, mouse weasels and field voles [20]. Attached ticks were collected from the captured small mammals and separately stored in test tubes at − 20 °C until morphological species identification [19, 21, 22]. Necropsy was performed with biometric data on the rodents’ body measurements in order to determine each small mammal’s age. Detailed information on the small mammals and tick and flea species is provided in [17, 23] and is partially given in Additional file 1: Tables S1 and S2. Fleas and mites were collected with tweezers from the fur during small mammal dissection. Fleas were then stored individually in 100 µl RNALater solution (Qiagen, Hilden Germany) until morphological identification under a stereomicroscope [24]. For each flea and tick species, species identification was performed and confirmed by PCR targeting of the 18s rRNA and 16s rRNA genes [25, 26]. Mites were mounted for at least 12 h on permanent microscopic slides using gum-chloral medium (Liquido de Swann) [27] and subsequently morphologically identified under a microscope at a magnification of 40× using standardized taxonomic keys [28].

### Statistical analysis

Prevalence (percentage of infested hosts among all screened hosts) was reported together with the Wilson score confidence interval (95% confidence level). Mean intensity (total number of ectoparasites divided by all infested hosts) was reported together with the standard error of the mean (SEM). Prevalence and mean intensity were combined to obtain mean abundance by multiplication as a quantitative descriptor of the ectoparasite population [29].

Regression analyses were conducted on two levels: on the group level for objective (ii) in which ectoparasite species were combined into three groups (ticks/mites/fleas) and on the species level for objective (iii), whereby individual ectoparasite species abundance was the outcome variable. Groups were formed by order or class (Siphonaptera or Acari, respectively), with the exception of mites, which were treated independently. Only ectoparasite species that occurred with an overall prevalence of > 5% were analyzed on the latter level. Regression analyses on both group and species level were only performed on hosts trapped in the forest and in the recreational area. Only one host species (wood mouse) was trapped in the urban location and nowhere else, leading to complete separation of this host species in the regression analysis. Twenty animals trapped in the recreational area that were not screened for mites were also excluded from regression analysis, as were animals with undetermined sex or age in the species level regression models.

Regression analysis was performed to assess the influence of location (forest/recreational area), host species (*A. flavicollis*/*M. glareolus*) and seasonality (spring/summer/autumn-winter) on the abundance of ectoparasite groups in group level models. Possible host-specific factors, such as sex, age (juvenile/young adult/adult), location, season and infestation with other ectoparasites, were analyzed on the species level with ectoparasite species abundance as the outcome variable.

Ectoparasite abundance data was modeled in a hurdle model, which allowed for the joint analysis of prevalence and mean intensity in one model [30]. Hurdle models are two-part models, with the first part modeling the prevalence (infested/not infested) in a logistic regression model (LR); once this hurdle is crossed, a truncated negative binomial model (TNB) with the mean intensity as a count variable is modeled. This two-step approach allows the prevalence and mean intensity of the ectoparasite groups to be determined for different parameters and allows for both excess zeros in the data and overdispersion, both of which are often present in ecological data, to be accommodated in the model. Overdispersion was assessed by comparing the dispersion of simulated residuals, calculated from synthetic datasets using the fitted models, to the observed residuals and inspecting quantile-quantile plots (QQ plots) and residual* versus* predicted values plots [31]. Zero-inflation was assessed by comparing inflated and non-inflated models in a Vuong test and by comparing the distribution of expected zeros to observed zeros [31]. Model selection was based on the Akaike information criterion (AIC) and the Bayesian information criterion (BIC) values of the models, starting with a full model and excluding variables from the model until the AIC und BIC values no longer decreased. The model with the lowest AIC/BIC ratio was chosen. If it was not possible to fit a hurdle model or if it was not the best fitting model compared to a generalized linear model, a Poisson or negative binomial model was chosen with abundance as the outcome variable. For ectoparasites occurring with a mean intensity of < 2, a logistic regression model was chosen and only the information on prevalence was used in the model.

When in some cases the two AIC and BIC gave conflicting results, the simpler model was chosen [30]. Model fit of the final model was inspected in a rootogram, where observed and predicted counts were visually compared [32]. For the hurdle models, the odds ratios of the binary logistic regression (LR-OR) and incidence rate ratios of the truncated negative binomial regression (TNB-IRR) with the corresponding* p* values and 95% confidence intervals (95% CI) are presented in the text and tables.

Season was included as a categorical variable. Spring was defined as the period March–May and summer as June–August. In the months between December and March, small mammal trapping took place only sporadically. For this reason, autumn and winter were combined into one category to include the months September–February. Polyparasitism is defined as the number of species infesting a host. It was treated as a discrete variable and was only considered in the species level models.

Age of the hosts was defined as a categorical variable with three levels based on the recorded weight of the trapped rodents. Categories were defined for each host species as: *A. flavicollis*: < 20 g, < 3.5 months old; 20–30 g, 3.5–7 months old; > 30 g, ≥ 7 months old; *M. glareolus*: < 15 g, < 1.5 months old; 15–19.5 g, 1.5–2.5 months old; > 19.5 g, ≥ 2.5 months old. Sex was included as a binary variable (male/female).

For comparison of ectoparasite prevalence between rodents in one trapping location or between host species, chi-squared tests or, alternatively, Fisher’s exact tests were performed for samples with small positive counts.

All statistical analyses were performed using R statistical software version 3.6.1 (The R Foundation for Statistical Computing, Vienna, Austria). The PropCIs package was used for Wilson score confidence intervals [33], the MASS, pscl and glmmTMB package for negative binomial and hurdle models [34–36]. The DHARMa package was used to assess dispersion and for the zero-inflation analysis [31], and the countreg package was used for rootograms [35]. Outcome tables were produced with the sjPlot package [37].

## Results

### Determination of species diversity

A total of 5691 ectoparasite specimens belonging to 27 species were collected from 775 small mammals. Species richness was the lowest in the urban location with 12 ectoparasite species, compared to 19 species in the forest and 20 in the recreational area (Additional file 1: Table S1). The ectoparasites within the class of Insecta belonged to the order Siphonaptera (fleas) comprising four families (Leptopsyllidae, Ceratophylliodea, Ctenophthalmidae and Hystrichopsyllidae) with 11 species. Within the class of Arachnida, subclass Acari, three species of ticks (order Ixodida; family Ixodidae), one species of the order Sarcoptiformes and nine species within four families (Euryparasitidae, Laelapidae, Macrochelidae and Hamogamasidae) of the order Mesostigmata were detected.

In total, 689 of 775 (88.9%; 95% CI 86.50, 90.93) small mammals were infested with ectoparasites, with an average of 7.34 (standard deviation [SD] 11.58) specimens per host. Hosts were infested with up to six species of ectoparasites simultaneously. An infestation with one or two ectoparasite species per host was most frequently observed, with 253 (32.6%) and 242 (31.2%) hosts infested with one or two species, respectively. *Apodemus flavicollis* and *A. agrarius* were more frequently infested with two ectoparasite species than with one, with 64 *A. flavicollis* (25.9%) infested with one ectoparasite species and 75 (30.4%) infested with two species. None of the four trapped *A. agrarius* were infested with only one species of ectoparasite, and three (75%) were infested with two species (Table 1).

**Table 1:** Number of ectoparasite species on different host species at three different locations in Germany

Number of ectoparasite species simultaneously infesting a single host	Number of ectoparasite species infesting a host species (percentage of infested host in group of all infested hosts)							
	*Apodemus sylv aticus* (*n* =36)	*Apodemus flavicollis* (*n* =247)	*Myodes glareolus* (*n* = 473)	*Apodemus agrarius* (*n* = 4)	*Sorex* spp. (*n* = 6)	*Microtus arvalis* (*n* = 8)	*Microtus agrestis* (*n* = 1)	All species (*n* = 775)
0	0 (0.0%)	12 (4.9%)	68 (14.4%)	0 (0.0%)	3 (50.0%)	2 (25.0%)	1 (100.0%)	86 (11.2%)
1	10 (27.7%)	64 (25.9%)	175 (37.0%)	0 (0.0%)	3 (50.0%)	1 (12.5%))	0 (0.0%)	253 (32.6%)
2	9 (25.0%)	75 (30.4%)	151 (31.9%)	3 (75.0%)	0 (0.0%)	4 (50.0%)	0 (0.0%)	242 (31.2%)
3	8 (22.2%)	75 (30.4%)	61 (12.9%)	1 (25.0%)	0 (0.0%)	1 (12.5%)	0 (0.0%)	146 (18.8%)
≥ 4	9 (25.0%)	21 (8.5%)	18 (3.8%)	0 (0.0%)	0 (0.0%)	0 (0.0%)	0 (0.0%)	48 (6.2%)

### Investigation of the effect of host species, trapping location and season on the infestation of small mammals with ectoparasites

 An overview of the prevalence of small mammals being affected with the three different ectoparasite groups and their mean intensity across the three trapping locations is provided in Table 2.

**Table 2:** Prevalence and mean intensity of ectoparasite groups in small mammals trapped at three different locations

Ectoparasite group	Urban (*n* = 36 hosts)		Forest (*n* = 241 hosts)		Recreational area (*n* = 498 hosts)	
	Prevalence (95% CI)	Mean intensity (SE)	Prevalence (95% CI)	Mean intensity (SE)	Prevalence (95% CI)	Mean intensity (SE)
Ticks	83.33% (68.11–92.13)	5.13 (0.87)	82.57% (77.28–86.84)	5.95 (0.64)	61.04% (56.69–65.23)	6.75 (0.73)
Mites	47.22% (31.99–62.99)	2.82 (0.66)	36.93% (31.09–43.18)	8.39 (1.18)	19.68% (16.42–23.40)	3.56 (0.43)
Fleas	66.67% (50.33–79.79)	4.04 (0.67)	61.83% (55.55–67.73)	2.64 (0.24)	57.63% (56.69–65.23)	2.31 (0.12)

CI, Confidence interval; SE, standard error

The prevalence of ticks was higher than that of mites or fleas. More than 80% of the hosts in urban and forest areas had ticks, while fleas were present in around 60% of the hosts and mites only in 20% of the hosts in the recreational area.

#### Mean intensity and prevalence of ticks

Ticks were the most frequently observed group of ectoparasites, with an overall prevalence of 68.77% (95% CI 65.43, 71.94). Three species of ticks were found during the study period. *Ixodes ricinus* was the most commonly observed species of ticks, with a prevalence of 68.52% (95% CI 65.16, 71.69) and was found in all three trapping locations and on all host species. *Ixodes trianguliceps* was exclusively found in the forest location, while *D. reticulatus* was only observed in the recreational area. Prevalence was low for both these species, ranging between 3.32% (95% CI 1.69, 6.41) and 3.01% (95% CI 1.83, 4.91), with no significant difference in infestation of *A. flavicollis* or *M. glareolus* (*I. trianguliceps*: Fisher test *P* = 0.634; *D. reticulatus*: Fisher test *P* = 0.289). The mean intensity of the infestation was 1.0 for all occurrences of *I. trianguliceps* and *D. reticulatus*, except for *M. glareolus* in the recreational area, which was infested with a mean intensity of 9.2 (SE 2.7) with *D. reticulatus.*

**Fig. 1:**
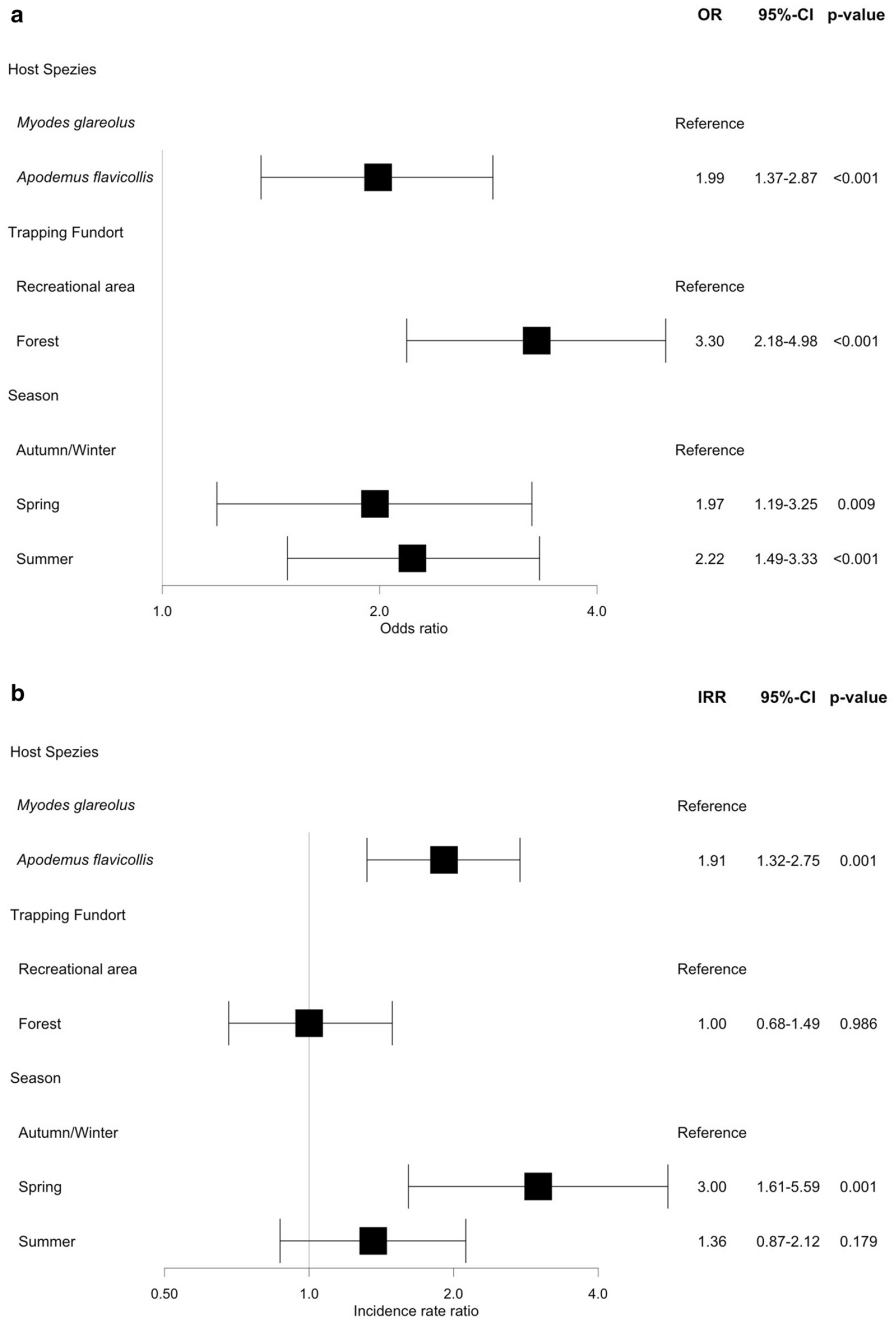
Parameters affecting the abundance of ticks on wild rodents from results using a hurdle model with prevalence (**a**) and mean intensity (**b**).* 95% CI* 95% confidence interval,* IRR* incidence rate ratio,* OR* odds ratio. Vertical gray line indicates no association, with OR = 1.0 or IRR = 1.0

Factors significantly influencing the prevalence of ticks in the forest location and the recreational area were host species, trapping location and season, and factors influencing the mean intensity were host species and season (spring) (Fig. 1). *Apodemus flavicollis* was more likely to be infested than *M. glareolus* (LR-OR 1.99, *P* < 0.001; TNB-IRR 1.91, *P* < 0.001). The season also significantly influenced the prevalence and the mean intensity of ticks on small mammals with spring and summer as the seasons with the highest odds to find ticks compared to winter (LR-OR: 1.97, *P* = 0.009 spring; 2.22, *P* < 0.001 summer). The mean intensity of ticks was highest during spring (TNB-IRR 3.00, *P* = 0.001) compared to winter, but was not statistically significantly higher during summer (TNB-IRR 1.36, *P* = 0.179). The trapping location did not significantly influence the mean intensity of ticks (TNB-IRR 1.00, *P* = 0.986), but it significantly influenced the prevalence of ticks, with small mammals in the forest being much more frequently infested than in the recreational area (LR-OR 3.30, *P* < 0.001).

#### Mean intensity and prevalence of mites

Mites had a prevalence of 26.32% (95% CI 23.34, 29.53), with the most prevalent family being Laelapidae (21.55%, 95% CI 18.80, 24.58). The most frequently observed species of mite was *L. agilis*, with a prevalence between 72.45% (95% CI 62.88, 80.32) and 42.28% (95% CI 34.64, 50.31) for *A. flavicollis* in the forest location and the recreational area. *Myodes glareolus* was only infested in the forest location, with a prevalence of 5.04% (95% CI 2.46, 10.03). *Laelaps agilis* also affected *Apodemus sylvaticus* in the urban location, but was not found on *A. agrarius*,* Sorex* spp. or *Microtus arvalis*. *Eulaelaps stabularis* was the second most frequently observed mite species, with a low prevalence in both the forest location (1.66%; 95% CI 0.65, 4.19) and recreational area (3.6%; 95% CI 2.21, 5.76) but a higher prevalence in the urban park (19.44%; 95% CI 9.75; 35.03). *Haemogamasus nidi* was also observed frequently and had the highest prevalence in the urban location (5.5%; 95% CI 1.54, 18.14). Other mite species were observed less frequently. Oribatid mites and *Androlaelaps farenholzi* were only found in the recreational area while *Haemogamasus hirsutosimilis* and *Euryparasitus emerginatus* were only found in the forest. Like ticks, mites were found on small mammals in all three trapping locations, but the prevalence significantly differed between locations (Chi^2^-test: 33.423, *P* < 0.001), with the lowest prevalence found in the recreational area and the highest in the urban area (Table 2). Only a few individuals belonging to the species *A. agrarius* and *M. arvalis* (*n* = 6 for each) were trapped in the recreational areas and these were not infested with mites at all (Additional file 1: Table S3). From the *Sorex* spp*.* trapped in the forest, one specimen was infested with mites (*Eur. emerginatus*).

**Fig. 2:**
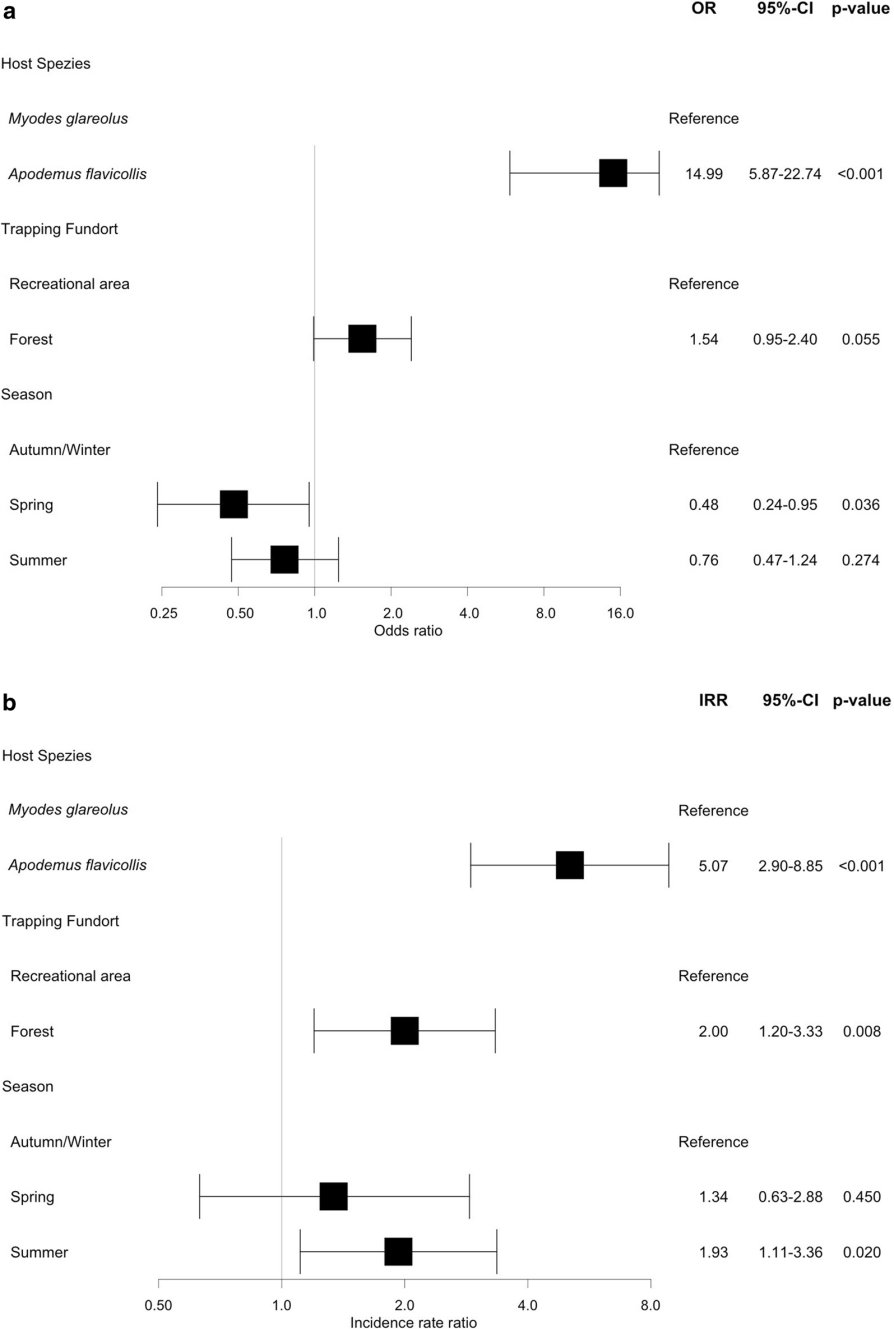
Parameters affecting the abundance of mites on wild rodents from results using a hurdle model with prevalence (**a**) and mean intensity (**b**). Gray line indicates no association with *OR* = 1.0 or *IRR* = 1.0. *OR* Odds ratio, *95% CI* Confidence Interval of 95% probability, *IRR* Incidence rate ratio

Factors influencing the prevalence of mites in the group level regression models were host species and season, with a higher prevalence for the host species being *A. flavicollis* and a lower prevalence for the season being spring (Fig. 2). The mean intensity was affected by host species, trapping location and season (summer). *Apodemus flavicollis* was more often infested than *M. glareolus* (LR-OR 14.99, *P* < 0.001) and with a much higher mean intensity (TNB-IRR 5.07, *P* < 0.001) (Fig. 2).

#### Mean intensity and prevalence of fleas

Fleas had an overall prevalence of 59.35% (95% CI 55.86, 62.76), with Ctenophthalmidae being the most prevalent family (51.87%; 95% CI 48.35, 55.37). The most prevalent species of fleas was *C. agyrtes*. *Apodemus flavicollis* in the forest location was slightly more often infested (66.33%; 95% CI 56.51, 74.91) than *M. glareolus* in the forest or the recreational area, which ranged between 48.20% (95% CI 40.06; 56.44) and 50.60% (95% CI 45.26; 55.92), respectively. The flea species *Me. turbidus* had a prevalence of 15.87% (95% CI 12.34; 20.17) in the recreational area and of 5.04% (95% CI 2.46; 10.03) in the forest on *M. glareolus*, whereas its prevalence on *A. flavicollis* was 5.37% (95% CI 2.75, 10.24) and 2.04% (95% CI 0.56, 7.14) for the forest location and recreational area, respectively.

**Fig. 3:**
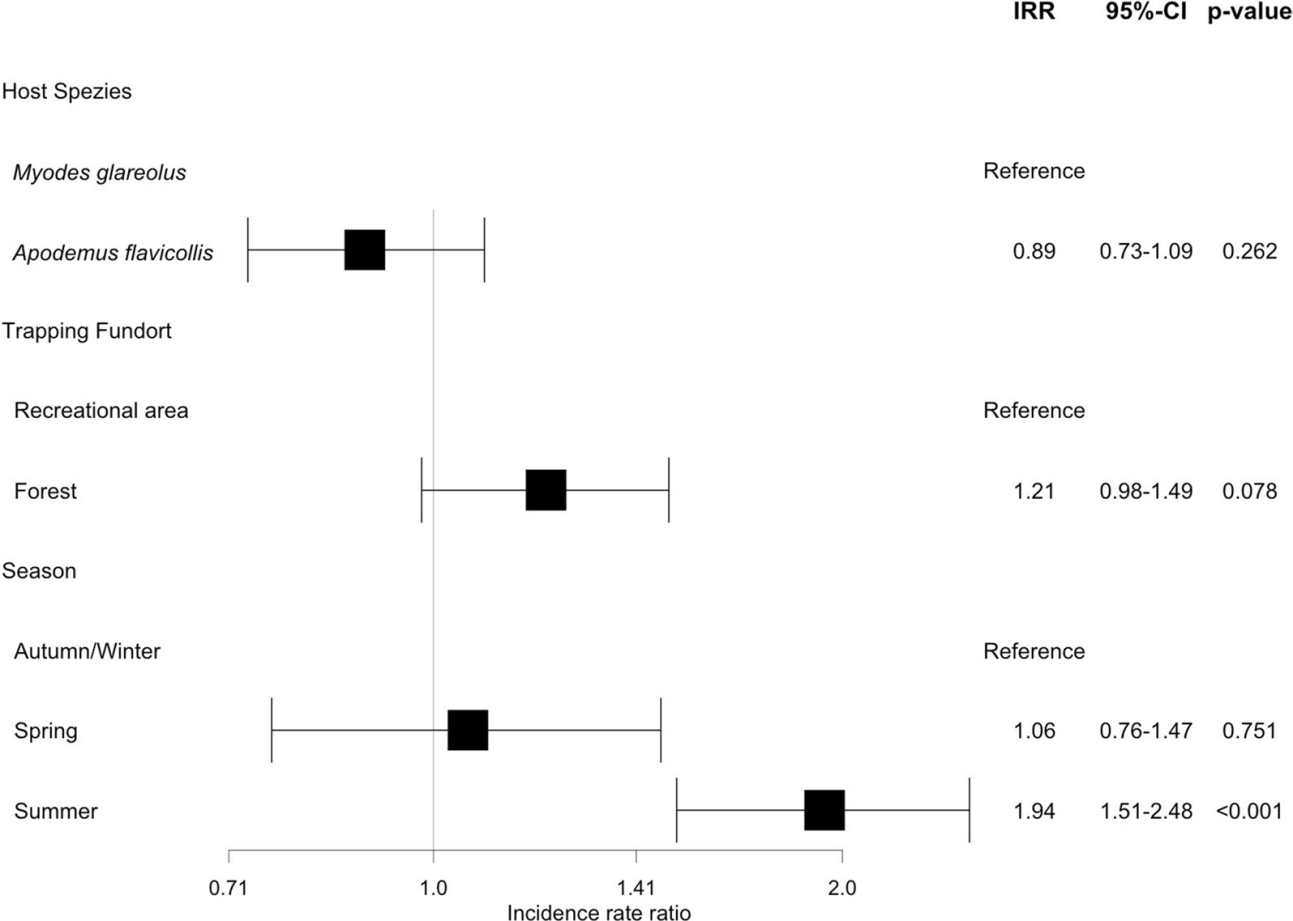
Parameters affecting the abundance of fleas on wild rodents trapped in Germany modeled in a negative binomial model. Gray line indicates no association with *IRR* = 1.0. *IRR* Incidence rate ratio, *95% CI* Confidence Interval of 95% probability

In the regression analysis no hurdle model could be fitted; therefore the abundance was modeled in a negative binomial model. The abundance of fleas was not significantly influenced by either host species or the location of trapping, but by season (NB-IRR spring 1.06, *P* = 0.751; NB-IRR summer 1.94, *P* < 0.001) (Fig. 3).

### Assessment of the parameters influencing the abundance of the most frequently observed ectoparasite species

*Ixodes ricinus*, *Laelaps agilis*, *Ctenophthalmus agyrtes* and *Megabothris turbidus* were each observed with an overall prevalence of > 5% and thus analyzed separately. Due to the low sample size of *Ctenophthalmus congener congener* and *C. bisoctodentatus*, these flea species were grouped and analyzed together.

*Ixodes ricinus* was more prevalent in the forest (LR-OR 3.19, *P* < 0.001) and during the spring and summer (LR-OR spring 2.99, *P* < 0.001; LR-OR summer 1.73, *P* = 0.024); however, *A. flavicollis* was not more likely to be infested by *I. ricinus* (Table 3) even though the host species had a significant influence on the mean intensity of *I. ricinus* (TNB-IRR 2.45, *P* < 0.001). Being infested with other ectoparasite species also had a strong influence on infestation with *I. ricinus* ticks, but it did not have an influence on the mean intensity of *I. ricinus*. Each additional infestation with an ectoparasite species was associated with an increase in the odds (4.76; *P* < 0.001) of being infested with *I. ricinus.* Being a male host or in a higher age category also significantly influenced the mean intensity of *I. ricinus* ticks (TNB-IRR-male 1.84, *P* < 0.001; TNB-IRR young adult 1.79, *P* = 0.034, TNB-IRR adult 1.76, *P* = 0.049).

**Table 3:** Parameters affecting the abundance of *Ixodes ricinus* ticks on small mammals in Germany according to factors influencing the prevalence and mean intensity

Factors affecting prevalence			
Predictors	OR	95% CI	*P *value
(Intercept)	0.09	0.05–0.16	< 0.001*
Trapping location			
Recreational area	Reference		
Forest	3.19	1.98–5.14	< 0.001*
Season			
Autumn/winter	Reference		
Spring	2.99	1.63–5.47	< 0.001*
Summer	1.73	1.07–2.78	0.024*
Polyparasitism	4.76	3.65–6.22	< 0.001*

Factors affecting mean intensity			
Predictors	IRR	95% CI	*P* value
(Intercept)	0.62	0.30–1.28	0.200
Host species			
*Myodes glareolus*	Reference		
* Apodemus flavicollis*	2.45	1.76–3.40	< 0.001*
Host sex			
Female	Reference		
Male	1.84	1.33–2.56	< 0.001*
Age category			
Juvenile	Reference		
Young adult	1.79	1.04–3.07	0.034*
Adult	1.76	1.00–3.09	0.049
Season			
Autumn/winter	Reference		
Spring	2.01	1.14–3.55	0.016*
Summer	0.97	0.64–1.47	0.888

IRR, Incidence rate ratio; OR, odds ratio

*Significant effect at* P* ≤ 0.05

*Laelaps agilis* mites were almost exclusively found on *A. flavicollis* (LR-OR 121.95, *P* < 0.001) with only five *M. glareolus* in the forest being infested with this species (Additional file 1: Table S3). Even though small mammals trapped in the forest did not have a higher prevalence of *L. agilis* compared to those in the recreational area, they did have a higher mean intensity (LR-OR 1.57, *P* = 0.173; TNB-IRR 1.92, *P* = 0.031) (Table 4). The same was true for male hosts (TNB-IRR 1.76, *P* = 0.029). Polyparasitism also had a statistically significant positive influence on the prevalence of *L. agilis* mites (LR-OR 3.07, *P* < 0.001). Hosts trapped in the spring had slightly lower odds to be infested with *L. agilis* than those trapped in the winter (LR-OR 0.23, *P* = 0.005). This association was not statistically significant for hosts trapped in the summer (LR-OR 0.79, *P* = 0.524).

**Table 4:** Parameters affecting the abundance of *Laelaps agilis* mites on small mammals in Germany with factors influencing the prevalence and the mean intensity

Factors affecting prevalence			
Predictors	OR	95% CI	*P* value
(Intercept)	0.00	0.00–0.01	< 0.001*
Host species			
*Myodes glareolus*	Reference		
*Apodemus flavicollis*	121.95	49.45–300.74	< 0.001*
Trapping location			
Recreational area	Reference		
Forest	1.57	0.82–2.99	0.173
Season			
Autumn/Winter	Reference		
Spring	0.23	0.08–0.64	0.005*
Summer	0.79	0.39–1.61	0.524
Polyparasitism	3.07	2.22–4.25	< 0.001*

Factors affecting mean intensity			
Predictors	IRR	95% CI	*P* value
(Intercept)	0.62	0.30–1.28	0.200
Host species			
*Myodes glareolus*	Reference		
*Apodemus flavicollis*	5.15	1.60–16.62	0.006*
Trapping location			
Recreational area	Reference		
Forest	2.18	1.22–3.92	0.009*
Host sex			
Female	Reference		
Male	1.76	1.06–2.93	0.029*
Season			
Autumn/Winter	Reference		
Spring	1.54	0.55–4.34	0.411
Summer	1.82	0.95–3.50	0.070

IRR, Incidence rate ratio; OR, odds ratio

*Significant effect at* P* ≤ 0.05

The prevalence of *C. agyrtes* was higher on *M. glareolus* and on hosts trapped during the summer (LR-OR *A. flavicollis* 0.35, *P* < 0.001; LR-OR summer 1.82, *P* = 0.015) and also for small mammals infested with multiple ectoparasite species, with each additional infestation associated with an increase in the odds of 6.74 (*P* < 0.001) (Table 5).

**Table 5:** Parameters affecting the abundance of *Ctenophthalmus agyrtes* fleas on small mammals in Germany with factors influencing the prevalence and the mean intensity

Factors affecting prevalence			
Predictors	OR	95% CI	*P* value
(Intercept)	0.05	0.02–0.09	< 0.001*
Host species			
* Myodes glareolus*	Reference		
* Apodemus flavicollis*	0.35	0.22–0.55	< 0.001*
Host sex			
Male	Reference		
Female	0.61	0.42–0.90	0.012*
Season			
Autumn/Winter	Reference		
Spring	1.64	0.90–3.02	0.108
Summer	1.82	1.12–2.94	0.015*
Polyparasitism	6.74	5.08–8.94	< 0.001*
**Factors affecting mean intensity**			
**Predictors**	**IRR**	**95% CI**	***P*** **value**
(Intercept)	0.23	0.10–0.56	0.001*
Trapping location			
Recreational area	Reference		
Forest	1.58	1.11–2.25	0.011*
Host sex			
Male	Reference		
Female	0.72	0.51–1.02	0.064
Season			
Autumn/winter	Reference		
Spring	1.74	0.91–3.34	0.094
Summer	2.76	1.68–4.52	< 0.001*
Polyparasitism	1.19	0.98–1.44	0.077

IRR, Incidence rate ratio; OR, odds ratio

*Significant effect at* P* ≤ 0.05

The prevalence of *Me. turbidus* was higher for *M. glareolus* (LR-OR *A. flavicollis* 0.13, *P* < 0.001) in the recreational area (LR-OR-forest 0.24, *P* < 0.001) and 3.62-fold as high for each additional infestation with an ectoparasite species (LR-OR polyparasitism 3.62, *P* < 0.001) (Table 6). The same factors statistically significantly affected the prevalence of *C. bisoctodentatus* and *C. congener congener* (LR-OR *A. flavicollis* 0.32, *P* = 0.008; LR-OR-forest 0.07, *P* < 0.001; LR-OR-polyparasitism 2.84, *P* < 0.001) (Table 7).

**Table 6:** Parameters affecting the abundance of *Megabothris turbidus* fleas on small mammals in Germany modeled in a negative binomial model

Predictors	OR	95% CI	*P* value
(Intercept)	0.01	0.00–0.03	< 0.001*
Trapping location			
Recreational area	Reference		
Forest	0.24	0.11–0.53	< 0.001*
Host species			
* Myodes glareolus*	Reference		
* Apodemus flavicollis*	0.13	0.06–0.31	< 0.001*
Season			
Autumn/Winter	Reference		
Spring	1.78	0.59–5.40	0.310
Summer	2.18	0.86–5.48	0.099
Polyparasitism	3.62	2.66–4.92	< 0.001*

IRR, Incidence rate ratio; OR, odds ratio

*Significant effect at* P* ≤ 0.05

**Table 7:** Parameters affecting the abundance of *Ctenophthalmus* (*C. congener* and *C. bisoctodentatus* analyzed jointly) fleas on small mammals in Germany modeled in a negative binomial model

Predictors	OR	95% CI	*P* value
(Intercept)	0.01	0.01–0.03	< 0.001*
Trapping location			
Recreational area	Reference		
Forest	0.07	0.02–0.31	< 0.001*
Host species			
* Myodes glareolus*	Reference		
* Apodemus flavicollis*	0.32	0.14–0.74	0.008*
Polyparasitism	2.84	2.08–3.87	< 0.001*

IRR, Incidence rate ratio; OR, odds ratio

*Significant effect at* P* ≤ 0.05

## Discussion

The burden of ectoparasites on small mammals serving as main hosts for many different ectoparasite species was analyzed in this study. In total, we found 27 different ectoparasite species of three important ectoparasite groups (fleas, ticks, mites) on eight small mammal species. These ectoparasites are also considered to be important vectors of arthropod-borne zoonotic pathogens. The existence of suitable host species as well as other environmental factors directly influence the local abundance of ectoparasites, and seasonal fluctuations of ectoparasite infestation must be regarded in context of host population dynamics [3]. For example, population sizes of *A. flavicollis* and *M. glareolus*, which fluctuate throughout the year and are the highest in late summer, may lead to a dilution effect on the mean intensities of ectoparasites per individual rodent [13]. Furthermore, polyparasitism in general leads to higher abundance as well as higher prevalence rates, which was observed for all of the most frequently collected ectoparasite species (*L. agilis*, *C. agyrtes*, *I. ricinus*), on small mammals in this study. This phenomenon may be explained by the resistance of individual mammalian hosts. Animals with a weaker immune system may attract more ectoparasites and thus more ectoparasite species. To analyze the key factors affecting the mean intensity and prevalence of different ectoparasite orders and species on their host, we established several statistical models in the present study.

A common phenomenon in ecological data when, for example, looking at species distributions or population dynamics are the excess zeros (= more zeroes in a data set than the distribution allows for). In this context, it is important to distinguish between “true zeros,” which are caused by an underlying mechanism, and “false zeros,” which are produced, for example, by errors in the data collection. Different regression models, such as the zero-inflated Poisson, negative binomial models or hurdle models, can be used to address this issue [38]. In this study, we assumed that only one mechanism produced counts of zero in the dataset, i.e. the true absence of infestation. Zero-inflated models assume two mechanisms to produce zero counts, whereas hurdle models do not have the underlying assumption that different mechanisms produce the zeros (in this study = no infestation with ectoparasites) present in the dataset [39]. All small mammals were trapped live, ensuring that ectoparasites were still present on the animal during species determination and, therefore, ensuring that no infestations were missed. It is important to note that an absence of an ectoparasite species on a trapped rodent does not equal the absence in the trapping location due to the ecology of the studied species. For example, ticks only stay on the host for feeding and then leave the host, while only adult fleas are parasitic.

The analysis of abundance in a hurdle model offers the additional advantage of distinguishing between factors influencing the prevalence and factors influencing the mean intensity by modeling both separately. However, because of the low occurrence of several ectoparasite species, data were grouped by order (ticks/mites/fleas) to achieve a sufficient sample size and to model abundance or otherwise risk non-convergence, as described in previous literature [3].

Ticks were the most frequently found ectoparasite group, having the highest infestation rate per small mammal species in comparison to mites and fleas. A possible reason for this relatively higher infestation rate may be that mites as well as fleas have a host preference which is host-specific or at least host-opportunistic [40]. In contrast, *D. reticulatus* and *I. ricinus* ticks have a broad host range thus can feed on different hosts. The tick species found in the current study were *D. reticulatus*, *I. ricinus* and *I. trianguliceps*. The tick species *I. ricinus* was by far the most prevalent ectoparasite in this study, parasitizing on six small mammal species. This tick species is known to have a very diverse host spectrum, which makes it the most important vector for tick-borne pathogens in central Europe [10]. In our study, *A. flavicollis* were infested with the highest mean intensity of this tick species; however, in earlier studies a similar host preference of *I. ricinus* was described [3, 41]. Furthermore, we observed in this study that not only *I. ricinus* ticks showed a clear host preference for *A. flavicollis*, but all other tick species did so as well. By being repeatedly infested with *I. ricinus* ticks, *M. glareolus* builds a resistance against *I. ricinus* larvae, leading to lower infestation rates [42], possibly explaining the lower infestation rate in comparison to *A. flavicollis* observed in the present study. *Ixodes trianguliceps*, a nidicolous tick species, parasitizes small mammals in preference to middle-sized or large mammals [43]. In particular, *Microtus* spp. are regarded as the main hosts of *I. trianguliceps*. However, in our study this tick species was found mostly on *M. glareolus,* which is also discussed as a possible host [43]. *Dermacentor reticulatus* has a focal distribution pattern in Germany and occurs mostly in south-west Germany and in a few areas in eastern Germany [44]. In the present study we found it only on small mammals from eastern Germany and not at the study sites from southeast Germany. It was predominantly found on *M. glareolus* in previous studies from Germany, which was confirmed by our results [18, 45]. One factor influencing the prevalence of ticks on small mammals was the trapping location. A similar result was reported by Maaz et al. [3] who also showed significant differences between trapping locations, with higher prevalence rates in forested areas compared to urban sites. A possible explanation for this may be the higher diversity and abundance of different host species in the forested area, such as roe deer or wild boar, which are not likely to be present at the urban site. The prevalence of ticks as well as the mean intensity in particular of *I. ricinus* were higher in the spring compared to all other seasons. *Ixodes ricinus* larvae were by far the most common developmental stage found on small mammals in this study. Questing larvae have an activity peak in the spring which may explain the higher prevalence and mean intensity in this season on small mammals [46]. The *I. ricinus* burden was higher on older and male individuals. A possible reason for the higher mean intensity in males may be that they have a broader activity range than females and thus may encounter ticks more frequently [47]. Further, the higher testosterone levels in males, associated with a higher stress level and therefore lower immunity, may favor tick infestation [48]. Previous studies showed a higher mean intensity of ticks on older rodents compared to younger individuals [49]. Our findings are in line with this finding, which was earlier explained by a correlation between age and body mass [1]. However, one should consider that age estimation was based on the weight of the small mammals. This method may have a slight distorting effect as weight discrepancies due to pregnancy, obesity or cachexia cannot be ruled out.

In this study, seven mite species belonging to the family Laelapidae were detected. In another study from Germany studying ectoparasites on small mammals, 13 mite species belonging to this family were detected [3]. The mean intensity was higher in summer and at the forest site. Predatory mites, such as *Ornithonyssus* spp. which are known to parasitize rats, develop quicker in a warmer (over 20 °C) and more humid climate [50]. This may explain the higher mean intensity of *L. agilis* in the summer and in the forest. *Laelaps agilis* was by far the most often detected mite species in this study, confirming results from earlier studies in Germany [3, 14]. Over 95% of *L. agilis* were detected on *Apodemus* spp. which are the preferred hosts of this mite species [51, 52]. Further, *L. agilis* was more likely to be found on male individuals, which may likewise be explained by their higher testosterone level and thus lower immunity, as already described for ticks. *Laelaps hilaris* mostly occurred on voles in the present study. These findings are in line with reports from previous studies in Germany and the Czech Republic showing the same host preferences [3, 52]. *Haemogamasus nidi, Eu. stabularis* and *L. agilis* are often encountered together on the same host (mostly *Apodemus* spp.) [53]. This was confirmed by our results. Moreover, it has been reported that *Haemogamasus hirsutosimilis* and *H. arvicolarum* occur only on *Apodemus* spp., which is likewise in line with our findings [52, 54]. Most of the mites mentioned here were found occasionally positive for *Coxiella burnetii*, *Francisella* spp., TBEV and *Rickettsia* spp. Nonetheless, these results do not necessarily reflect a vector function, as positive mites may have just recently picked up a blood meal from a pathogen-positive host. Only one specimen of *Androlaelaps fahrenholzi* was found in the present study. Previous studies reported this mite species on different small mammal species but also on birds [55, 56, 56] and even on humans [57].

Oribatid mites, *Eur. emarginatus* and *Ma. glaber* are non-parasitic arthropods. Oribatida are soil mites feeding on dead or alive plants and are most probably only accidentally collected from a rodent [58]. *Macrocheles* spp. are known to be phoretic in small mammals’ fur but not parasitic [59]. *Euryparasitus emarginatus* may occur on voles occasionally [60]; however this mite species is known to be nidicolous and prefers to live in the nests of small mammals (mostly shrews and moles) rather than on the small mammals themselves [61].

The diversity of flea species in this study was higher than the diversity seen for ticks or mites and also higher than that observed in a recent study on fleas from small mammals in Germany [3]. *Typhloceras poppei*, *Nosopsyllus fasciatus*, *C. congener congener*, *C. bisoctodentatus* as well as *Hystrichopsylla talpae talpae* are known to parasitize voles such as *M. glareolus* and mice such as *Apodemus* spp., which is confirmed by our results [60]. However, the above-mentioned flea species may also be associated with dogs and cats and thus are possible vectors for zoonotic pathogens [62]. *Ctenophthalmus agyrtes* has a broad host range and may be found on most small mammals in Europe [63]. In our study it was the most prevalent flea species, occurring at all study sites and on five different small mammal species belonging to three different genera. It was more frequently found on *M. glareolus* compared to *A. flavicollis* and in the sylvatic study site Moreover, this flea species is regarded as harboring zoonotic bacteria, such as *Bartonella* spp. and *Rickettsia* spp. [63, 64]. *Paleopsylla soricis* and *Peromyscopsylla sylvatica* are adapted to shrews; however they may also occur on bank voles [65]. In the present study we detected these flea species almost exclusively on bank voles. However, the shrews investigated in this study were found dead in the traps. This is why it is not surprising that none of the shrews was infested with fleas, as it is known that fleas leave their hosts as soon as the body temperature begins to drop [66]. *Megabothris walkeri* and *Me. rectangulatus* are mainly associated with voles [65]. In our study, *Me. walkeri* occurred likewise on the field vole as well as on the bank vole and not on mice. *Megabothris turbidus* is a commensal flea species on both voles and mice. In our study, it could be predominantly found on *M. glareolus,* which is in line with previous findings by our group [64]. It was mostly found at the recreational area. In general, the recreational study site had the highest species diversity. This study site was renatured in the early 2000s, making it a new unestablished area, which may be the reason for the high species diversity. Unestablished areas such as this renatured site usually have a relatively higher species diversity due to yet unestablished ecological niches with intraspecific as well as interspecific competitors [67]. *Leptopsylla segnis* may be a vector of *Rickettsia typhi*, which is the causative agent of the murine typhus [68, 69]. This flea species is common on the house mouse (*Mus musculus*) and the Norway rat (*Rattus norvegicus*), which are known to live close to human settlements. It may also occasionally occur on the wood mouse (*A. sylvaticus* [70]). In our study, this flea species was exclusively found on *A. sylvaticus* and at the urban site, with close contact to human settlements and, thus, the house mouse. *Nosopsyllus fasciatus* is likewise a flea species which is known to infest mostly Norway rats. This flea species was likewise found at the urban site but was also detected at both of the other sites.

A previous study reported a higher mean flea intensity on *Apodemus* spp. than on voles, which was explained by the larger body size of *Apodemus* spp. in comparison to voles [3]. This observation was also significant in the present study. Previous studies have reported a seasonal variation of flea infestation with highest infestation rates from April-September in comparison to winter [71]. These results are in line with the findings from our group reporting higher infestation rates in summer. However, one should consider that in this study, the trapping of small mammals did not take place in some winter months due to bad weather conditions.

## Conclusions

To conclude, nearly 90% of small mammals were infested with ectoparasites with up to six different ectoparasite species found on one host simultaneously. Most remarkably, the diversity of flea species found was unexpectedly high and higher than in comparable studies. The high diversity could be detected due to the differently structured habitats (urban, renatured, sylvatic) of the regions examined in this study. Further, polyparasitism was a key influencing factor, with a positive effect on the prevalence and/or abundance of the mainly occurring ectoparasite species *I. ricinus*, *L. agilis*, *Me. turbidus* and *C. agyrtes* found on small mammals. Season can be regarded as an influencing factor on the mean intensity for ticks, mites and fleas, with either spring or summer being the most favorable. The trapping location had impact on the mean intensity and prevalence of ectoparasites, with the sylvatic site favored over the renatured site. While *Apodemus* spp. were more often infested with *I. ricinus* and *L. agilis*, *M. glareolus*, in comparison, was more often infested with *D. reticulatus* and *Me. turbidus*. The sex of the small mammals had an influence on the mean intensity of ticks and mites, but not on fleas, with male small mammals being at a higher risk. This study shows the key factors which influence the abundance of vectors. Since most ectoparasite species in this study, especially flea and tick species, are vectors of zoonotic pathogens with a broad range of mammal hosts, this study may make an important contribution to our understanding and prevention of vector-borne pathogens.

## Supplementary Information

**Additional file 1.**

## Data Availability

All data generated or analyzed during this study are included in this published article and its additional files.

## Abbreviations

*A.*: *Apodemus*
AIC: Akaike information criterion
BIC: Bayesian information criterion
*C.*: *Ctenophthalmus*
*D.*: *Dermacentor*
*Eu.*: *Euryparasitus*
*H.*: *Haemogamasus*
*I.*: *Ixodes*
IRR: Incidence rate ratio
*L.*: *Laelaps*
LR: Logistic regression model
*M.*: *Myodes*
*Me.*: *Megaborthris*
QQ plot: Quantile-quantile plot
*R.*: *Rickettsia*
TBEV: Tick-borne encephalitis virus
TNB: Truncated negative binomial model
