# Supplementary material for: Host–parasite interactions of rodent hosts and ectoparasite communities from different habitats in Germany
Source: Parasit Vectors. 2021 Feb 17;14:112. doi: 10.1186/s13071-021-04615-7 (PMC7890891; doi:10.1186/s13071-021-04615-7)
Supplement: Supplementary file 1 — Additional file 1. [file 13071_2021_4615_MOESM1_ESM.docx]

**Table S1.** Number of specimens of an ectoparasite species found on small rodents in three locations in Germany between 2012 and 2013.

| **Ectoparasite species** | **Location** | | | **Total** |
| --- | --- | --- | --- | --- |
|  | **Urban** | **Forest** | **Recreational area** |  |
| *Ixodes ricinus* | 154 | 1177 | 1932 | 3263 |
| *Ixodes trianguliceps* | - | 8 | - | 8 |
| *Dermacentor reticulatus* | - | - | 121 | 121 |
| Oribatida | - | 1 | - | 1 |
| *Euryparasitus emarginatus* | - | 2 | - | 2 |
| *Laelaps hilaris* | - | 40 | 33 | 73 |
| *Macrocheles glaber* | - | 4 | 2 | 6 |
| *Laelaps agilis* | 28 | 684 | 272 | 984 |
| *Haemogamasus hirsutosimilis* | - | 1 | 1 | 2 |
| *Haemogamasus nidi* | 2 | 10 | 21 | 33 |
| *Androlaelaps fahrenholzi* | - | - | 1 | 1 |
| *Eulaelaps stabularis* | 16 | 4 | 19 | 39 |
| *Haemogamasus arvicolarum* | 1 | - | - | 1 |
| *Vulgarogamasus* spp. | - | 1 | - | 1 |
| *Leptopsylla segnis* | 17 | - | - | 17 |
| *Megabothris turbidus* | 2 | 11 | 82 | 95 |
| *Ctenophthalmus agyrtes* | 50 | 340 | 483 | 873 |
| *Ctenophthalmus bisoctodentatus* | - | - | 3 | 3 |
| *Typhloceras poppei* | 7 | - | 4 | 11 |
| *Megabothris walkeri* | - | 6 | 4 | 10 |
| *Nosopsyllus fasciatus* | 20 | 4 | 19 | 43 |
| *Hystrichopsylla talpae talpae* | 1 | 4 | 4 | 9 |
| *Paleopsylla soricis* | - | 1 | 1 | 2 |
| *Peromyscopsylla silvatica* | - | 26 | 2 | 28 |
| *Ctenophthalmus congener congener* | - | 2 | 59 | 61 |
| *Megabothris rectangulatus* | - | - | 3 | 3 |

**Table S2.** Descriptive statistics of small rodents trapped in three locations in Germany during the years 2012 and 2013.

| Location | Small mammal species | | | | | | | | | | | | | | | | | | | | | | | | |
| --- | --- | --- | --- | --- | --- | --- | --- | --- | --- | --- | --- | --- | --- | --- | --- | --- | --- | --- | --- | --- | --- | --- | --- | --- | --- |
|  | *Apodemus sylvaticus* | | | *Apodemus flavicollis* | | | *Myodes glareolus* | | | *Apodemus agrarius* | | | *Sorex* spp. | | | *Microtus arvalis* | | | *Microtus agrestis* | | | Other | Total | | |
|  | n* | Sex (m/f)** | Mean Weight in g (sd)*** | n* | Sex (m/f)** | Mean Weight in g (sd)*** | n* | Sex (m/f)** | Mean Weight in g (sd)*** | n* | Sex (m/f)** | Mean Weight in g (sd)*** | n* | Sex (m/f)** | Mean Weight in g (sd)*** | n* | Sex (m/f)** | Mean Weight in g (sd)*** | n* | Sex (m/f)** | Mean Weight in g (sd)*** | n* | n* | Sex (m/f)** | Mean Weight in g (sd)*** |
| Urban | 36 | 28/7**** | 22.19 (5.25) | - | - | - | - | - | - | - | - | - | - | - | - | - | - | - | - | - | - | - | 36 | 28/7 | 22.19 (5.25) |
| Recreational area | - | - | - | 149 | 78/71 | 28.09 (6.45) | 334 | 185/148* | 19.45 (4.74) | 4 | 1/3 | 16.94 (4.36) | 2 | 0/1* | 8.19 (2.67) | 8 | 4/4 | 24.50 (7.51) | 1 | 1/0 | 18.02 (-) | 3 | 501 | 269/22 | 22.05 (6.71) |
| Forest | - | - | - | 98 | 46/52 | 25.31 (5.18) | 139 | 64/75 | 19.34 (4.19) | - | - | - | 4 | 2/2 | 9.92 (2.36) | - | - | - | - | - | - | 1 | 242 | 112/129 | 21.61 (5.64) |
| Total | 36 | 28/7 | 22.19 (5.25) | 247 |  |  | 473 |  |  | 4 | 1/3 | 16.94 (4.36) | 6 |  |  | 8 | 4/4 | 24.50 (7.51) | 1 | 1/0 | 18.02 (-) | 4 | 779 | 409/363 | 21.92 (6.33) |

*number of animals

** male/ female

*** standard deviation

****one specimen with undetermined sex

**Table S3.** Distribution of ectoparasite species on small mammal species on *Apodemus sylvaticus*, *Apodemus flavicollis* and *Myodes glareolus*.

| Ectoparasites found on small mammals | Small mammal hosts | | | | | | | | | | | | | | | | | | | |
| --- | --- | --- | --- | --- | --- | --- | --- | --- | --- | --- | --- | --- | --- | --- | --- | --- | --- | --- | --- | --- |
|  | *Apodemus sylvaticus* | | | | *Apodemus flavicollis* | | | | | | | | *Myodes glareolus* | | | | | | | |
|  | Urban | | | | Forest | | | | Recreational area | | | | Forest | | | | Recreational area | | | |
|  | n* | Prev. ** (%) | 95% CI*** | mI (SE)**** | n* | Prev. ** (%) | 95% CI*** | mI (SE)**** | n* | Prev. ** (%) | 95% CI*** | mI (SE)**** | n* | Prev. ** (%) | 95% CI*** | mI (SE)**** | n* | Prev. ** (%) | 95% CI*** | mI (SE)**** |
| All tick species | 30 | 83.33 | 68, 92 | 5.13 (0.87) | 96 | 97.96 | 93, 99 | 8.25 (1.22) | 97 | 65.10 | 57, 72 | 9.20 (1.85) | 101 | 72.66 | 65, 79 | 3.80 (0.37) | 199 | 59.58 | 54, 65 | 5.60 (0.64) |
| *Ixodes ricinus* | 30 | 83.33 | 68, 92 | 5.13 (0.87) | 96 | 97.96 | 93, 99 | 8.23 (1.22) | 96 | 64.43 | 56, 72 | 9.27 (1.86) | 101 | 72.66 | 65, 79 | 3.74 (0.37) | 198 | 59.28 | 54, 64 | 5.03 (0.59) |
| *Ixodes trianguliceps* | - | - | - | - | 2 | 2.04 | 1, 7 | 1.00 (0.00) | - | - | - | - | 6 | 4.32 | 2, 9 | 1.00 (0.00) | - | - | - | - |
| *Dermacentor reticulatus* | - | - | - | - |  | - | - | - | 2 | 1.34 | 0, 5 | 1.00 (0.00) | - | - | - | - | 13 | 3.89 | 2, 7 | 9.15 (2.66) |
| All mite species | 17 | 47.22 | 32, 63 | 2.82 (0.66) | 74 | 75.51 | 66, 83 | 9.61 (1.36) | 67 | 44.97 | 37, 53 | 4.34 (0.60) | 14 | 10.07 | 6, 16 | 2.50 (1.00) | 31 | 9.28 | 7, 13 | 1.87 (0.28) |
| *Haemogamasus arvicolarum* | 1 | 2.78 | 0, 14 | 1.00 | - | - | - | - | - | - | - | - | - | - | - | - | - | - | - | - |
| *Laelaps agilis* | 9 | 25.00 | 14, 41 | 3.11 (1.18) | 71 | 72.45 | 63, 80 | 9.32 (1.38) | 63 | 42.28 | 35, 50 | 4.32 (0.62) | 7 | 5.04 | 2, 10 | 3.14 (1.98) | - | - | - | - |
| *Eulaelaps stabularis* | 7 | 19.44 | 9, 35 | 2.29 (0.47) | 3 | 3.06 | 1, 9 | 1.00 (0.00) | 10 | 6.71 | 4, 12 | 1.10 (0.10) | 1 | 0.72 | 0, 4 | 1.00 | 6 | 1.80 | 1, 4 | 1.33 (0.33) |
| *Haemogamasus nidi* | 2 | 5.56 | 2, 18 | 1.00 (0.00) | 1 | 1.02 | 0, 6 | 5.00 | 5 | 3.36 | 1, 8 | 1.40 (0.40) | 4 | 2.88 | 1, 7 | 1.25 (0.25) | 11 | 3.29 | 2, 6 | 1.27 (0.27) |
| Oribatida*.* | - | - | - | - | - | - | - | - | - | - | - | - | 1 | 0.72 | 0, 4 | 1.00 | - | - | - | - |
| *Haemogamasus hirsutosimilis* | - | - | - | - | 1 | 1.02 | 0, 6 | 1.00 | 1 | 0.67 | 0, 3 | 1.00 | - | - | - | - | - | - | - | - |
| *Euryparasitus emarginatus* | - | - | - | - | 1 | 1.02 | 0, 6 | 1.00 | - | - | - | - | - | - | - | - | - | - | - | - |
| *Laelaps hilaris* | - | - | - | - | 4 | 4.08 | 2, 10 | 8.75 (6.45) | - | - | - | - | 1 | 0.72 | 0, 4 | 5.00 | 13 | 3.89 | 2, 7 | 2.54 (0.58) |
| *Macrocheles glaber* | - | - | - | - | 1 | 1.02 | 0, 6 | 4.00 | - | - | - | - | - | - | - | - | 1 | 0.30 | 0, 2 | 2.00 |
| *Androlaelaps fahrenholzi* | - | - | - | - | - | - | - | - | - | - | - | - | - | - | - | - | 1 | 0.30 | 0, 2 | 1.00 |
| Other | 1 | 2.78 | 1, 14 | 1.00 | - | - | - | - | - | - | - | - | 1 | 0.72 | 0, 4 | 1.00 | - | - | - | - |
| All flea species | 24 | 66.67 | 50, 80 | 4.04 (0.67) | 69 | 70.41 | 61, 79 | 2.87 (0.46) | 74 | 49.66 | 42, 58 | 1.93 (0.14) | 80 | 57.55 | 49, 65 | 2.45 (0.23) | 203 | 60.78 | 55, 66 | 2.44 (0.15) |
| *Megabothris turbidus* | 2 | 5.56 | 1, 18 | 1.00 (0.00) | 2 | 2.04 | 1, 7 | 1.00 (0.00) | 8 | 5.37 | 3, 10 | 1.00 (0.00) | 7 | 5.04 | 2, 10 | 1.29 (0.29) | 53 | 15.87 | 12, 20 | 1.34 (0.08) |
| *Ctenophthalmus agyrtes* | 16 | 44.44 | 30, 60 | 3.13 (0.57) | 65 | 66.33 | 57, 75 | 2.89 (0.46) | 63 | 42.28 | 35, 50 | 1.60 (0.11) | 67 | 48.20 | 40, 56 | 2.27 (0.25) | 169 | 50.60 | 45, 56 | 2.15 (0.15) |
| *Ctenophthalmus bisoctodentatus* | - | - | - | - | - | - | - | - | 3 | 2.01 | 1, 6 | 1.00 (0.00) | - | - | - | - | - | - | - | - |
| *Typhloceras poppei* | 2 | 5.56 | 1.5, 18 | 3.50 (1.50) | - | - | - | - | 2 | 1.34 | 0, 5 | 1.50 (0.50) | - | - | - | - | 1 | 0.30 | 0, 2 | 1.00 |
| *Megabothris walkeri* | - | - | - | - | 2 | 2.04 | 1, 7 | 1.00 (0.00) |  | - | - | - | 2 | 1.44 | 0, 5 | 2.00 (1.00) | 2 | 0.60 | 0, 2 | 1.00 (0.00) |
| *Nosopsyllus fasciatus* | 12 | 33.33 | 20, 50 | 1.67 (0.33) | 2 | 2.04 | 1, 7 | 1.00 (0.00) | 12 | 8.05 | 5, 14 | 1.42 (0.23) | 2 | 1.44 | 0, 5 | 1.00 (0.00) | 2 | 0.60 | 0, 2 | 1.00 (0.00) |
| *Hystrichopsylla talpae talpae* | 1 | 2.78 | 0.5, 14 | 1.00 | 2 | 2.04 | 1, 7 | 1.00 (0.00) | 2 | 1.34 | 0, 5 | 1.00 (0.00) | 2 | 1.44 | 0, 5 | 1.00 (0.00) | 2 | 0.60 | 0, 4 | 1.00 (0.00) |
| *Paleopsylla soricis* | - | - | - | - | - | - | - | - | - | - | - | - | 1 | 0.72 | 0, 4 | 1.00 | 1 | 0.30 | 0, 2 | 1.00 |
| *Peromyscopsylla silvatica* | - | - | - | - | 1 | 1.02 | 0, 6 | 1.00 | - | - | - | - | 22 | 15.83 | 11, 23 | 1.14 (0.07) | 2 | 0.60 | 0, 2 | 1.00 (0.00) |
| *Ctenophthalmus congener congener* | - | - | - | - | 1 | 1.02 | 0, 6 | 1.00 | 5 | 3.36 | 1, 81 | 1.60 (0.60) | 1 | 0.72 | 0, 4 | 1.00 | 34 | 10.18 | 7, 14 | 1.44 (0.15) |
| *Megabothris rectangulatus* | - | - | - | - | - | - | - | - | 1 | 0.67 | 0, 4 | 1.00 | - | - | - | - | 1 | 0.30 | 0, 2 | 2.00 |
| *Leptopsylla segnis* | 10 | 27.78 | 16, 44 | 1.70 (0.30) | - | - | - | - | - | - | - | - | - | - | - | - | - | - | - | - |

* number of animals

** prevalence

*** confidence interval

**** mean intensity with standard error

Table S4. Distribution of ectoparasite species on small mammal species on *Microtus arvalis*, *Apodemus agrarius*, *Sorex* spp. and *Microtus agrestis*.

| Ectoparasite species found on small mammals | Small mammal hosts | | | | | | | | | | | | | | | | | | | |
| --- | --- | --- | --- | --- | --- | --- | --- | --- | --- | --- | --- | --- | --- | --- | --- | --- | --- | --- | --- | --- |
|  | *Microtus arvalis* | | | | *Apodemus agrarius* | | | | *Sorex* spp. | | | | | | | | *Microtus agrestis* | | | |
|  | Recreational area | | | | Recreational area | | | | Forest | | | | Recreational area * | | | | Recreational area * | | | |
|  | n* | Prev. **(%) | 95% CI*** | mI (SE)**** | n* | Prev. **(%) | 95% CI*** | mI (SE)**** | n* | Prev.** (%) | 95% CI*** | mI (SE)**** | n* | Prev.** (%) | 95% CI*** | mI (SE)**** | n* | Prev. (%)** | 95% CI*** | mI (SE)**** |
| All tick species | 4 | 50.0 | 22, 78 | 4.25 (1.44) | 4 | 100 | 51, 100 | 7.25 (5.60) | 2 | 50 | 15, 85 | 4.5 (0.5) | - | - | - | - | - | - | - | - |
| *Ixodes ricinus* | 4 | 50.0 | 22, 78 | 4.25 (1.44) | 4 | 100 | 51, 100 | 7.25 (5.60) | 2 | 50 | 15, 85 | 4.5 (0.5) | - | - | - | - | - | - | - | - |
| *Ixodes trianguliceps* | - | - | - | - | - | - | - | - | - | - | - | - | - | - | - | - | - | - | - | - |
| *Dermacentor reticulatus* | - | - | - | - | - | - | - | - | - | - | - | - | - | - | - | - | - | - | - | - |
| All mite species | - | - | - | - | - | - | - | - | 1 | 25 | 5, 70 | - | - | - | - | - | - | - | - | - |
| *Haemogamasus arvicolarum* | - | - | - | - | - | - | - | - | - | - | - | - | - | - | - | - | - | - | - | - |
| *Laelaps agilis* | - | - | - | - | - | - | - | - | - | - | - | - | - | - | - | - | - | - | - | - |
| *Eulaelaps stabularis* | - | - | - | - | - | - | - | - | - | - | - | - | - | - | - | - | - | - | - | - |
| *Haemogamasus nidi* | - | - | - | - | - | - | - | - | - | - | - | - | - | - | - | - | - | - | - | - |
| Oribatida. | - | - | - | - | - | - | - | - | - | - | - | - | - | - | - | - | - | - | - | - |
| *Haemogamasus hirsutosimilis* | - | - | - | - | - | - | - | - | - | - | - | - | - | - | - | - | - | - | - | - |
| *Euryparasitus emarginatus* | - | - | - | - | - | - | - | - | 1 | 25 | 5, 70 | - | - | - | - | - | - | - | - | - |
| *Laelaps hilaris* | - | - | - | - | - | - | - | - | - | - | - | - | - | - | - | - | - | - | - | - |
| *Macrocheles glaber* | - | - | - | - | - | - | - | - | - | - | - | - | - | - | - | - | - | - | - | - |
| *Androlaelaps fahrenholzi* | - | - | - | - | - | - | - | - | - | - | - | - | - | - | - | - | - | - | - | - |
| Other | - | - | - | - | - | - | - | - | - | - | - | - | - | - | - | - | - | - | - | - |
| All flea species | 6 | 75.0 | 41, 93 | 3.00 (0.97) | 4 | 100 | 51, 100 | 1.75 (0.75) | - | - | - | - | - | - | - | - | - | - | - | - |
| *Megabothris turbidus* | 1 | 12.5 | 2, 47 | 2.00 | 1 | 25 | 5, 70 | 1 | - | - | - | - | - | - | - | - | - | - | - | - |
| *Ctenophthalmus agyrtes* | 4 | 50.0 | 22, 78 | 3.25 (1.11) | 3 | 75 | 30, 95 | 1.67 (0.67) | - | - | - | - | - | - | - | - | - | - | - | - |
| *Ctenophthalmus bisoctodentatus* | - | - | - | - | - | - | - | - | - | - | - | - | - | - | - | - | - | - | - | - |
| *Typhloceras poppei* | - | - | - | - | - | - | - | - | - | - | - | - | - | - | - | - | - | - | - | - |
| *Megabothris walkeri* | 2 | 25.0 | 7, 59 | 1.00 (0.00) | - | - | - | - | - | - | - | - | - | - | - | - | - | - | - | - |
| *Nosopsyllus fasciatus* | - | - | - | - | - | - | - | - | - | - | - | - | - | - | - | - | - | - | - | - |
| *Hystrichopsylla talpae talpae* | - | - | - | - | - | - | - | - | - | - | - | - | - | - | - | - | - | - | - | - |
| *Paleopsylla soricis* | - | - | - | - | - | - | - | - | - | - | - | - | - | - | - | - | - | - | - | - |
| *Permyscopsylla silvatica* | - | - | - | - | - | - | - | - | - | - | - | - | - | - | - | - | - | - | - | - |
| *Ctenophthalmus congener congener* | 1 | 12.5 | 2, 47 | 1.00 | 1 | 25 | 5, 70 | - | - | - | - | - | - | - | - | - | - | - | - | - |
| *Megabothris rectangulatus* | - | - | - | - | - | - | - | - | - | - | - | - | - | - | - | - | - | - | - | - |
| *Leptopsylla segnis* | - | - | - | - | - | - | - | - | - | - | - | - | - | - | - | - | - | - | - | - |

* number of animals

** prevalence

*** confidence interval

**** mean intensity with standard error
